# How does growth mindset affect mental health of high school students during the COVID-19 epidemic? The role of grit and coping strategies

**DOI:** 10.3389/fpsyt.2022.969572

**Published:** 2022-09-20

**Authors:** Libin Zhang, Huan Qi, Chenxu Wang, Tian Wang, Yunyun Zhang

**Affiliations:** ^1^Collaborative Innovation Center of Assessment Toward Basic Education Quality, Beijing Normal University, Beijing, China; ^2^College of Psychology and Mental Health, North China University of Science and Technology, Tangshan, China

**Keywords:** growth mindset, grit, cope strategies, mental health, adolescents, COVID-19

## Abstract

**Background:**

The outbreak of COVID-19 epidemic continues to unfold globally, which harms the public’s mental health. Adolescents’ mental health is affected by social isolation and lockdown during the COVID-19 epidemic. The implicit theory of thoughts-emotion-behavior states that individuals with a growth mindset believe that thoughts, emotions, and behaviors can be changed through effort and tend to persist in pursuing higher goals and maintain enthusiasm as well as cope with stress resiliently, thus having higher gritty and levels of mental health. This study aimed to explore the role of grit and coping strategies in the influence of the growth mindset on adolescents’ mental health during the COVID-19 epidemic period.

**Methods:**

A total of 1564 participants (*M*_age_ = 17.02, 760 boys, 804 girls) from three high schools in China were recruited to complete The Self-report Questionnaire-20, The Growth Mindset Scale, The Short Grit Scale, and The Coping Style Scale to evaluate mental health, growth mindset, grit, and positive coping strategies, respectively.

**Results:**

The results showed that growth mindset has no significant indirect effect on mental health through grit but has a significant indirect effect on mental health through coping strategies. The results of chain mediation analysis showed that grit and coping strategies play chain mediating roles between growth mindset and adolescents’ mental health.

**Conclusion:**

The findings suggest that cultivating a growth mindset, developing grit, and teaching adolescents to adopt positive coping strategies can improve adolescents’ mental health.

## Introduction

The COVID-19 epidemic has swept the globe in recent years (1). Blockade measures prevent the spread of COVID-19 and benefit physical health, but ensuing social isolation also had a negative impact on mental health (2). Since the first emergence of the neo-crown virus in 2019, how to stop the spread of the virus while maintaining a healthy level of psychological wellbeing in people become a hot research topic in recent years.

### Growth mindset and mental health

The Implicit Theories of Intelligence (ITI) divided mindset into a growth mindset and fixed mindset (3). Based on the ITI, a new model called the implicit theory of thoughts, emotions, and behaviors (TEB) combines TEB, which are closely connected to mental health (4). Based on exploratory factor analysis (5) and confirmatory factor analysis (6), TEB were found as three independent but related factors. Existed study also demonstrated that a fixed mindset of TEB among adolescents in grades 6–8 was associated with an increase in their mental health problems (5). Specifically, a growth mindset can reduce the impact of family stress on externalizing behavior (4). To date, there are few studies on the implicit theory of TEB, but research on it is promising, more research is needed.

The growth mindset is a capacity for adaptation and change (7), which not only facilitates academic progress (8), but also plays a crucial role in overcoming social adversity, improving students’ social skills, and buffering adolescents from externalizing problems caused by family pressure (9). Research has shown that growth mindset can reduce adolescents’ depression caused by cyberbullying (10). Even for people with illnesses, enhancing growth mindset during treatment can increase their wellbeing (11). Research manifested that people with a growth mindset are committed to pursuing challenges, value effort, and are able to cope with setbacks in a positive way, thereby maintaining their mental health (12), thus moving forward by affirming the possibility of their future development and success. These conclusions indicate that growth mindset can, to some extent, reduce the negative emotions and improve adolescents’ mental health. Therefore, this study hypothesized that H1: growth mindset has a positive effect on the mental health of Chinese adolescents.

### Grit

Positive psychology states that individuals who follow a meaningful life exhibit higher levels of grit, which is a future-oriented effort and persistence of interest. Grit supports individuals’ pursuit of long-term goals, and it has two meanings, namely, perseverance and consistency of interest (9). Grit has also been shown to have a beneficial effect on mental health by reducing loneliness in life during the COVID-19 epidemic (13).

On the one hand, as a personality and basic psychological characteristic, grit predict an individual’s subjective wellbeing. An intervention study found that motivating college students’ grit enhance their satisfaction with realistic needs as well as their subjective experience (14). On the other hand, grit has the quality of responsibility, which enables individuals to become resilient in the face of adversity and failure (15), more importantly, keep the enthusiasm to be progressive (16, 17).

In addition, a study of Chinese children found that growth mindset predicted grit positively (18). Cultivating growth mindset may be particularly relevant to preventing or reducing mental health problems in adolescents’ development. Therefore, this study hypothesized H2: growth mindset has a positive impact on the mental health of the Chinese adolescent during the COVID-19 epidemic through the mediating role of grit.

### Coping strategy

Coping strategies is the individuals’ cognitive assessment of the stressful situation, and the relationships between individuals and the stressful situations are constantly changing in response to changes in the individuals’ behaviors (19). Positive coping strategies is solution-focused, aiming to do something to change the source of stress. Negative-coping strategies, focusing on emotions to avoid direct confrontation with stress situation and indirectly reducing emotional tension (20).

Researcher noted that United States undergraduate students who apply positive coping strategies and view stressors as potential challenges rather than threats, have lower perceptions of stress (21). Likewise, a Chinese study revealed similar results that mature, positive coping strategies can alleviate Chinese graduate students’ abnormal psychological symptoms arising in stressful situations (22). A study of disease groups found that people with multiple sclerosis who used positive problem-solving coping strategies had a higher level of mental health (23).

Adolescents are inevitable to take online teaching due to the lockdown during the COVID-19 epidemic. Negative-coping strategies resulting by lack of contact with peers and teachers, as well as low frequency physical activity are associated with poor mental health conditions (24). Researchers noted that as important predictors of mental health, positive coping strategies contribute to cope with the deterioration of mental health status due to the COVID-19 epidemic (25). Those who use negative coping strategies, such as emotion-focused strategies, tend to use alcohol, tobacco, and drugs (26) to relieve stress, which is at greater risk for adverse outcomes and can cause significant harm to mental health than those who rely on positive coping strategies (27).

In light of this, the present study hypothesized that H3: growth mindset has a positive impact on Chinese adolescents’ mental health during the COVID-19 epidemic through the mediating effect of positive coping strategy.

### Grit and coping strategy

**FIGURE 1:**
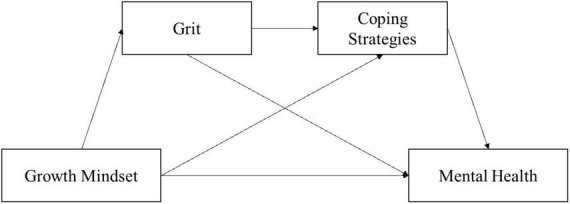
Hypothetical indirect pathways between growth mindset and adolescents’ mental health.

The perception of uncontrollable stressful events triggers higher stress and negatively affects emotional wellbeing (28). Existing research indicated that people with higher grit tend to adopt effective coping strategies to reduce perceived stress (29), which is benefit to alleviate fear and anxiety, thereby, maintain high levels of mental health (30). Based on TEB, this study hypothesized that H4: grit and coping strategy play a chain-mediated role in the influence of growth mindset on Chinese adolescents’ mental health. The theoretical model is shown in Figure 1.

## Materials and methods

### Participants

In this study, 1,596 students were recruited from three high schools in China. In total, thirty-two students were missing since they were absent on the day of data collection, or other undisclosed personal reasons. As a result, 1,564 students (760 men; 804 women; M_age_ = 17.02 years, *SD* = 1.05) were identified as final participants of this study, which resulted in an effective rate of 97. 99%. These include 539 senior students, 523 sophomores, and 502 juniors. There were 640, 406, and 518 students from urban, suburban, and rural areas, respectively. The questionnaires were distributed in the classes and would be collected after the survey was completed. This study was approved by the Ethics Committee of North China University of Science and Technology. All the participation in this study was voluntary and informed consent was obtained from parents in advance.

### Measures

#### Self-report questionnaire 20

The mental health was evaluated by the self-report questionnaire 20 (SQR-20) which was published by WHO (31). The Chinese revision of this scale has stable reliability and validity (32). In developing countries, SRQ-20 is a simple rapid screening tool for mental disorders, each item is scored on a two-point scale for “yes” or “no.” Higher scores indicate more prominent symptoms of psychiatric disorders. When the score is higher than 8, the individual is in a stressed state should be concerned. The Cronbach’s α coefficient of the scale was 0.916 in the present study.

#### Growth mindset scale

The Growth Mindset Scale (GMS) developed by Brock and Hundley (33) was used in this study to assess students’ growth mindset. GMS consists of 10 items and was rated on 2-point scale (–1 = “yes,” 1 = “no”). Higher scores of the total score indicates higher of growth mindset, conversely, lower score indicates higher fixed mindset. The Cronbach’s α coefficient of the scale was 0.744 in the present study. Confirmatory factor analysis showed that the construct validity of the GMS was good in this study, χ^2^/*df* = 7.285, CFI = 0.950, TLI = 0.931, RMSEA = 0.063, SRMR = 0.040.

#### The short grit scale

The Short Grit Scale (Grit-S) (34) was used in this study to evaluate the grit of participants, which included two dimensions, namely consistency of interest and perseverance of effort. Grit-S consists of eight items on a 5-points scale, with internal consistency reliability assessed between 0.73 and 0.83 in the previous studies (35). Due to there were high correlation between the total score of grit and the two dimensions’ score, only the total score of grit was performed in the subsequent analysis. Higher scores indicate higher levels of grit. The Cronbach’s α coefficient of the scale was 0.720 in the present study. Confirmatory factor analysis showed that the construct validity of the Grit-S scale was good in this study, χ^2^/*df* = 5.664, CFI = 0.988, TLI = 0.981, RMSEA = 0.055, SRMR = 0.020.

#### The coping style scale

The coping strategies was evaluated using the coping style scale (TCSS) developed by Xie based on ways of copingquestionnaire (36, 37). TCSS is scored on a 4-point scale, with the positive-coping subscale consisting of 12 items and the negative-coping subscale consisting of 8 items. The positive-coping score minus the negative-coping score is the individuals’ coping propensity score. A higher score indicates that an individual has adopted more positive coping strategies. The Cronbach’s α coefficient of the scale was 0.869 in the present study.

### Data analysis

First, the Harman’s single factor test was used to validate for common method variance (38). Second, descriptive statistics for the variables were presented, followed by the Pearson correlation analysis between these variables, and gender difference tests for each variable. Third, the bootstrapping resampling method was used to analyze the mediating model in this study, which was a robust method for obtaining CIs for specific indirect effect under most conditions through taking a sample of size n cases with replacement from the original sample (39). All the statistical analyses were carried out using SPSS 28.0 (SPSS Inc., Chicago, Illinois, United States), of which PROCESS was used to analyze the chain mediating model by bootstrapping with 5,000 resamples to derive the 95% CIs.

### Common method biases

Since growth mindset, grit, coping strategies, and mental health were measured by self-report, which may cause potential common method bias in the study. The Harman’s single factor analysis manifested that the first factor in our data explained only 19.13% of the variance. As this value was lower than the critical cut-off point of 40% (40), indicating that the common method bias was negligible.

## Results

### Descriptive statistics of variables

The gender differences test in growth mindset, grit, coping strategies, and mental health were shown in Table 1. The results showed that there were significant gender differences in grit and mental health. Levels of grit and mental health in boys were significantly higher than those of girls. Mental health scores were grouped with a cut-off point of 8. It was found that 681 individuals (43.54%) were in a stressful state (289 boys, 392 girls) and 883 non-stressed individuals (471 boys, 412 girls). The results of the χ^2^-tests showed that the stress level has significant gender differences, with girls having a significantly higher proportion than boys.

**TABLE 1:** The gender difference in the key variables.

Variables	Boys (*N* = 760)	Girls (*N* = 804)	*t/*χ*^2^*	*p*	*Cohen d*
Growth mindset	0.57 ± 5.54	0.2 ± 5.21	1.355	0.176	0.069
Consistency of interest	12.34 ± 3.92	11.99 ± 3.96	1.751	0.080	0.089
Perseverance of effort	12.44 ± 3.99	12.04 ± 4.08	1.970	0.049	0.100
Grit	24.78 ± 5.35	24.02 ± 5.39	2.766	0.006	0.140
Coping strategies	14.94 ± 7.71	15.39 ± 7.81	–1.143	0.253	0.058
Mental healthy	6.46 ± 5.76	7.81 ± 5.8	–4.601	<0.001	0.233
Non-stressed state	471	412	18.297	<0.001	–
Stressed-state	289	392			

M, mean; SD, Standard Deviation.

The preliminary analyses of the study variables were shown in Table 2. The results indicated that there was a significant negative correlation between growth mindset, grit, coping strategies, and mental health (*rs* = –0.467 ∼ –0.273, *p*_*s*_ < 0.01), and also a significant positive correlation between growth mindset, grit, and coping strategies (*rs* = 0.296 ∼ 0.374, *p*_*s*_ < 0.01).

**TABLE 2:** Bivariate correlations for study variables.

	1	2	3	4	5	6	7	8
1. Gender (boys = 0, girls = 1)	1							
2. Age	0.060*	1						
3. Mental healthy	0.116**	0.039	1					
4. Growth mindset	–0.034	–0.029	–0.363**	1				
5. Grit	–0.070**	–0.031	–0.273**	0.330**	1			
6. Consistency of interest	–0.044	0.011	–0.112**	0.248**	0.664**	1		
7. Perseverance of effort	–0.050*	–0.052*	–0.255**	0.197**	0.684**	–0.091**	1	
8. Coping strategies	0.029	–0.097**	–0.467**	0.296**	0.374**	0.096**	0.405**	1

*p < 0.05; **p < 0.01.

### Analysis of direct and indirect pathways of growth mindset and adolescents’ mental health

**FIGURE 2:**
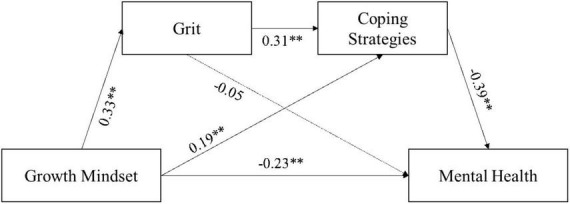
Estimates from chain mediation model testing the direct and indirect pathways between growth mindset and mental health. Estimates are standardized coefficients. The dashed line is the non-significant path. **p < 0.01.

As can be seen in Figure 2 and Table 3, the chain mediation model evaluated the direct and indirect pathways of growth mindset and adolescents’ mental health during the COVID-19 epidemic after controlling for the covariates of age and gender. In Model 1, the results revealed that growth mindset significantly predicted mental health negatively (β = –0.36, *p* < 0.01). In Model 2, the results revealed that growth mindset significantly predicted grit positively (β = 0.33, *p* < 0.01), growth mindset (β = 0.19, *p* < 0.01), and grit (β = 0.31, *p* < 0.01) significantly predicted coping strategies positively. In Model 3, the results revealed that growth mindset (β = –0.23, *p* < 0.01) and coping strategies (β = –0.39, *p* < 0.01) significantly predicted mental health negatively, and grit was not a significant predictor of mental health (β = –0.05, *p* < 0.01).

**TABLE 3:** The chain mediating model analysis between growth mindset and mental health.

	Model 1		Model 2				Model 3	
	Mental health		Grit		Coping strategies		Mental health	
	β	t	β	t	β	t	β	t
Constant	–0.313	–4.211**	0.171	2.241*	–0.182	–2.483*	–0.35	–5.221**
Age	0.023	0.964	–0.018	–0.759	–0.086	–3.742**	–0.014	–0.632
Gender	0.102	4.339**	–0.058	–2.404*	0.063	2.720**	0.117	5.425**
Growth mindset	–0.358	–15.272**	0.327	13.710**	0.193	7.947**	–0.230	–9.946**
Grit					0.312	12.854**	–0.045	–1.872
Coping strategies							–0.387	–16.377**
*R*2	0.143		0.113		0.184		0.289	
*F*	86.479**		65.934**		87.839**		126.815**	

*p < 0.05; **p < 0.01.

The mediating effect was measured by Bootstrapping for 5,000 resamples. As represented in Table 4, the total indirect effect of growth mindset on mental health was significant [β = –0.128,95%CI = (–0.155, –0.103)]. The indirect effect of growth mindset on mental health through grit was non-significant [β = –0.013, 95%CI = (–0.030, 0.0004)]. The indirect effect of growth mindset on mental health through coping strategies was significant [β = –0.075, 95% CI = (–0.097, –0.054)] accounting for 21.13% of the total effect. The chain mediated effect of growth mindset on mental health through grit and coping strategies was significant [β = –0.040, 95%CI = (–0.051, –0.029)] accounting for 11.27% of the total effect. The statistical results showed that grit and coping strategies play mediating roles in the chain of growth mindset influencing adolescents’ mental health.

**TABLE 4:** Standardized direct and indirect paths between growth mindset and adolescents’ mental health during COVID-2019.

Paths	β	SE	LLCI	ULCI	Percentage of effect size
**Total effect**	–0.355	0.023	–0.400	–0.309	–
**Direct effect**					
Growth mindset– > mental health	–0.227	0.023	–0.272	–0.182	–
**Total indirect effect**	–0.128	0.013	–0.155	–0.103	–
Growth mindset– > grit – > mental health	–0.013	0.008	–0.030	0.0004	3.66%
Growth mindset – > coping strategies – > mental health	–0.075	0.011	–0.097	–0.054	21.13%
Growth mindset – > grit– > coping strategies – > mental health	–0.040	0.006	–0.051	–0.029	11.27%

## Discussion

Because of the blockade during the COVID-19 epidemic adopted by various countries and regions, people were socially distanced, hindering the need for social interaction (41) and thus affecting the levels of mental health (42). For adolescents during the COVID-19 epidemic, the blockade strategy made it necessary to participate in online classes without face-to-face interaction with teachers and peers, which put a lot of stress on the psychological level and may even damage social confidence (43), at the same time, they are also under a tremendous psychological pressure.

Data from this study revealed that the Chinese adolescents who hold a growth mindset have fewer mental health problems and higher levels grit. At the same time, adolescents applying more coping strategies could reduce their mental health problems.

TEB theory states that a growth mindset is more beneficial to adolescents’ mental health than a fixed mindset (44). More importantly, a longitudinal study has shown that growth mindset is a negative predictor of adolescents’ mental health problems after 2 years (45), which means that the impact of growth mindset on adolescents’ mental health is stable over time. In the face of adversity, individuals with a growth mindset tend to believe that their abilities will develop over time, that effort is effective, and to be more active and flexible in applying the process of reaching achievement. Results of this study are consistent with previous research findings that growth mindset predicts greater grit in the face of social exclusion or victimization (46). As found in this study, Chinese adolescents who hold growth mindset adopt more coping strategies in facing difficulties and have less mental health problems. Meanwhile, in the process of overcoming various challenges, they will continue to improve the level of grit, therefore, they have higher levels of grit to apply more coping strategies to reduce mental health problems.

TEB implicit theory suggests that growth mindset aims to shape individuals’ thoughts, emotion, and behavior (4). Existed study demonstrated that negative coping strategies plays a negative role in regulating students’ mental health (47), which is negatively associated with mental health (48).

Growth-mind predisposes Chinese adolescents to maintain enthusiasm for pursuing goals and become gritter in the learning process, while there is a negative correlation between grit and depression and anxiety (49). In particular, during the COVID-19 epidemic, individuals with moderate and high levels of grit experienced less loneliness and academic stress resulting from social isolation (50), this is because they will exhibit more cognitive coping and actively seek support. Growth mindset makes Chinese adolescents more resilient when faced with challenges in life and academic. They refuse to give up and are willing to invest time and effort in trying various methods to overcome challenges and enjoy the joy of achieving success. Even if they fail, they will keep the same attitude to face the next challenge, while growth mindset makes individuals have more self-efficacy and maintain a high level of mental health. Meanwhile, growth mindset facilitates Chinese adolescents to regulate their emotions, adjust their behavior, and self-motivate to maintain a high level of mental health when facing and solving difficulties in life and school. Positive and flexible coping strategies brought by the growth mindset plays a great significance in improving the Chinese adolescents’ mental health. Therefore, the role of growth mindset in enhancing the Chinese adolescents’ mental health by improving grit and positive coping strategies should be emphasized.

This research contributes to future theoretical research and practical applications.

First, this study explores the impact of a growth mindset on the mental health among Chinese adolescents, and identifies the role of grit and coping strategies. In the future, based on the TEB and the results of this study, researchers can not only explore the influence of more thoughts, emotions and behaviors to enrich the research results of TEB, but also explore the relationship and impact pathway of growth mindset, grit, coping strategies, and mental health in different time period. It is worth noting that different ethnic groups of adolescents are necessary to research.

Second, in the future, more attention should be paid to the impact of growth mindset on the mental health of Chinese adolescents in school education, as well as the role of individuals’ grit and positive coping strategies. Along with teaching academics, school education should focus on and strengthen the growth mindset of students to keep high development levels while reducing the incidence of mental health problems in schools. Furthermore, our study also supports related psychological interventions studies.

Third, in addition to school education, this research also provides practical application values for parents and their children in the family environment. In daily life and family education, parents should realize that the importance of their children’s mental health and the cultivation of flexible cogitation of their children. Their children should be guided to solve life problems with flexible and diverse approach. For adolescents, fully understand the implication of growth mindset is conducive to improve their levels of grit, put forward a variety of solutions when facing problems, and thus maintaining a high level of mental health.

In addition, several limitations also exist. First, self-reported scales used in the present study cannot completely avoid social approval effects and common method bias. Therefore, so on-site observation and video recording may be considered in the future. Second, based on the current spread of the COVID-19 epidemic, we should consider exploring the long-term effects of growth mindset on adolescents’ mental health. Thus, longitudinal studies could be applied in the future research. It is worth mentioning that because the participants were randomly selected from three schools, caution was maintained in generalizing the results of the study. The samples could be further expanded to improve the validity of the results in future studies. Finally, the COVID-19 epidemic is now concentrated in some regions, while in counties and cities without the epidemic, normal work and life have resumed in each area. Therefore, in future studies, samples from areas with outbreaks can be compared with those from areas without outbreaks to explore whether the impact of the COVID-19 outbreak on adolescents’ growth mindsets is more profound in areas with concentrated epidemics.

## Conclusion

During the COVID-19 epidemic, adolescents faced tremendous psychological and adaptive stress. Those adolescents with growth mindset were able to maintain grit to pursue their goals, constantly adjust their ways of coping with the effects of the COVID-19 epidemic, and then maintain a healthy psychological level through positive coping strategies to handle the stress from school, life, and family.

## Data availability statement

The original contributions presented in this study are included in the article/supplementary material, further inquiries can be directed to the corresponding author.

## Ethics statement

The studies involving human participants were reviewed and approved by the Ethics Committee of North China University of Science and Technology. Written informed consent to participate in this study was provided by the participants’ legal guardian/next of kin.

## Author contributions

LZ: conceptualization, data—analysis and visualization, writing—original draft and review, and writing—editing. HQ: investigation, resources, data curation, methodology, and writing—original draft. CW and TW: writing—review and writing—editing. YZ: writing—review, supervision, and project administration. All authors contributed to the article and approved the submitted version.
